# Synchronous quadruple multiple primary cancers of the tongue, bilateral breasts, and kidney in a female patient with a disease-free survival time of more than 5 years: a case report

**DOI:** 10.1186/s12957-015-0684-5

**Published:** 2015-08-28

**Authors:** Tessho Maruyama, Toshiyuki Nakasone, Nobuyuki Maruyama, Akira Matayoshi, Akira Arasaki

**Affiliations:** Department of Oral and Maxillofacial Functional Rehabilitation, Graduate School of Medicine, University of the Ryukyus, 207 Uehara, Nishihara, Okinawa 903-0215 Japan; Department of Oral and Maxillofacial Surgery, University Hospital of the Ryukyus, 207 Uehara, Nishihara, Okinawa 903-0215 Japan; Departments of Dentistry and Oral Surgery, Chubu Tokushukai Hospital, 3-20-1, Teruya, Okinawa 904-8585 Japan

**Keywords:** Synchronous, Quadruple primary cancer, Oral, Surgery

## Abstract

**Background:**

Reports of synchronous multiple primary cancers in patients with oral cancer have recently been increasing because of progress in radiographic diagnostic techniques. Multiple primary cancers in patients with oral cavity cancer mainly occur in the head and neck region, lung, and esophagus. 2-[18F]-fluoro-2-deoxy-d-glucose positron emission tomography is usually used to identify synchronous multiple primary cancers.

**Case presentation:**

We herein describe a 69-year-old woman diagnosed with synchronous quadruple multiple primary cancers, namely a squamous cell carcinoma of the mobile tongue, invasive ductal carcinoma of the right breast, intraductal carcinoma of the left breast, and chromophobe renal cell carcinoma of the right kidney. We removed the four tumors over three surgical procedures to reduce the surgical risk because the patient had diabetes mellitus. To the best of our knowledge, this combination of multiple primary cancers has not been reported to date. Importantly, we followed this case for 5 years after surgery. The patient was alive and well with no clinical or radiologic signs of recurrent or metastatic disease at the time of this writing.

**Conclusions:**

In the present case, the kidney cancer could not be detected by 2-[18F]-fluoro-2-deoxy-d-glucose positron emission tomography but could be detected by contrast-enhanced computed tomography. To avoid overlooking multiple primary cancers of the kidney, we suggest that contrast-enhanced computed tomography should cover a region extending to the inferior margin of the kidney, rather than only to the liver, in patients with oral cavity cancer.

## Background

Multiple primary cancers (MPCs) are defined as more than two cancers detected in an individual patient. MPCs can be classified as either synchronous or metachronous according to their timing of diagnosis [1–4]. Reports of synchronous MPCs in patients with oral cancer have been increasing because of recent progress in radiographic diagnostic techniques. MPCs in patients with oral cavity cancer mainly occur in the head and neck region, lung, and esophagus (HNLE) [5, 6]. 2-[18F]-fluoro-2-deoxy-d-glucose positron emission tomography (FDG-PET) is usually used to identify synchronous MPCs [7, 8].

We herein report a case involving a patient with synchronous quadruple MPCs, namely a squamous cell carcinoma (SCC) of the tongue, ductal carcinoma of the bilateral breasts, and chromophobe renal cell carcinoma (RCC) of the right kidney. The kidney cancer could not be detected by FDG-PET but could be detected by contrast-enhanced computed tomography (CT).

## Case presentation

**Fig. 1:**
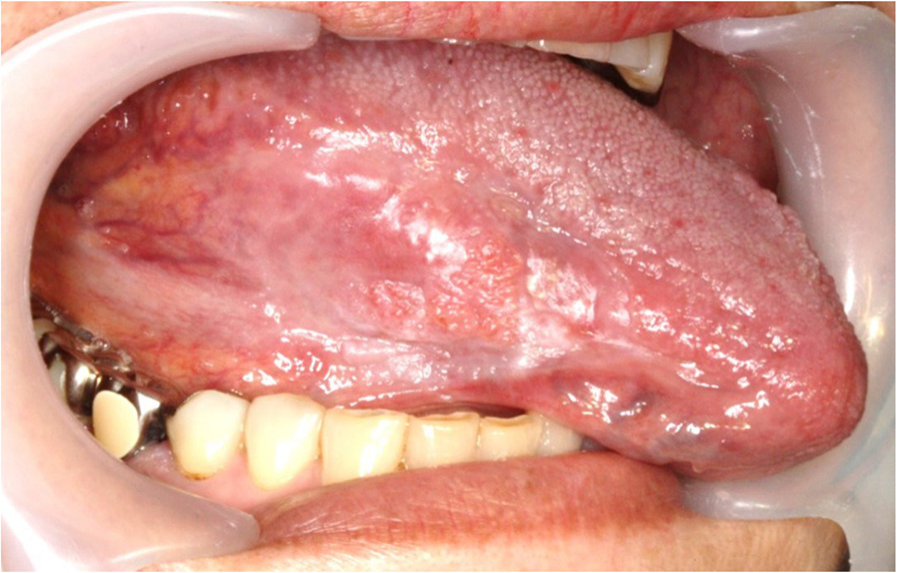
Physical examination revealed an elastic, hard, 1.2 × 0.7 cm mass of the right tongue

A 69-year-old woman was referred to our institute for further evaluation of a tongue mass. She had a 5-year history of pain involving the right lateral tongue edge, and the lesion had been diagnosed as lichen planus by incisional biopsy performed by her dentist. The disease was stable for a long time; however, the tongue pain suddenly worsened. Physical examination revealed an elastic, hard, 1.2 × 0.7 cm mass of the right tongue (Fig. 1). There was no palpable lymphadenopathy in the head and neck area. The patient was being medically treated for hypertension and diabetes mellitus, both of which were well controlled. She denied using tobacco or alcohol but had been exposed to smoke from her family. She had no history of exposure to ionizing radiation or having undergone cancer chemotherapy. Regarding her family history, her younger brother had been treated for rectal cancer and liver metastasis.

**Fig. 2:**
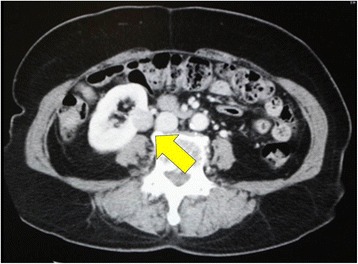
Renal cancer was suspected based on the CT findings (*arrow*)

Contrast-enhanced CT scans of the head and neck, chest, and liver were performed as a routine procedure in patients with oral cavity cancer. CT showed no lesions in the tongue, cervical lymph nodes, lungs, bone, or liver; however, three masses were detected in both breasts and the right kidney. Whole-body FDG-PET was performed to identify any other lesions; no other lesions were found. An upper gastrointestinal examination, indirect laryngoscopic examination, and bone scintigraphy revealed no abnormalities. The patient had no breast- or kidney-related signs or symptoms before we identified the masses. Fine-needle aspiration cytology was performed to confirm the diagnosis of the bilateral breast masses and revealed malignancy on both sides. Renal cancer was suspected based on the CT findings (Fig. 2, arrow). All four lesions were diagnosed as early-stage cancers at the initial presentation.

We removed the four tumors over three surgical procedures because the patient had diabetes mellitus. The patient underwent wide local excision of the tongue tumor. Histopathological examination revealed a moderately differentiated SCC. The bilateral breast lesions were then resected, and histopathological examination revealed an invasive ductal carcinoma (positive for estrogen receptor and human epidermal growth factor receptor 2, negative for progesterone receptor and MIB-1; 7–8 %) of the right breast representing a scirrhous growth with one metastatic axillary lymph node. An intraductal carcinoma of the left breast (MIB-1, 7–8 %) was also found. After resection of these lesions, laparoscopically assisted partial resection of the right kidney tumor was performed. Histopathological examination revealed a chromophobe RCC with positive cytoplasmic Hale’s colloidal iron staining. Immunohistochemistry, cytokeratin 7, CD10, and E-cadherin were positive.

In summary, we resected four synchronous MPCs including a moderately differentiated SCC of the mobile tongue, invasive ductal carcinoma of the right breast, intraductal carcinoma of the left breast, and chromophobe RCC of the right kidney. The patient received adjuvant therapy (radiation therapy and anastrozole) for both breast cancers. After a 5-year follow-up period, the patient was alive and well with no clinical or radiologic signs of recurrent or metastatic disease.

This case illustrates two important clinical issues. This combination of MPCs, namely that involving the tongue, bilateral breasts, and kidney, has not been reported to date. To avoid overlooking kidney MPC, we suggest that contrast-enhanced CT should cover an extended region ranging from the liver to the inferior margin of the kidney in patients with oral cavity cancer.

First, regarding the fact that this combination of MPCs has not been reported to date, we searched PubMed and the Japan Medical Abstracts Society databases but found no case reports of the same combination of cancers. The current case was defined as MPCs according to the Warren and Gates criteria [9]; namely, each cancer must be distinct, and the probability of one being a metastasis of the other must be excluded. Using this definition, all four neoplasms were determined to be primary cancers. Furthermore, we defined these cancers as synchronous cancers based on Moertel’s definition; that is, a primary tumor recognized within or after 6 months of diagnosis of another primary tumor is defined as synchronous or metachronous, respectively [1]. We thus diagnosed the present case as synchronous quadruple MPCs. We believe that the patient had three risk factors that contributed to this combination of MPCs. First, the patient had a previous diagnosis of diabetes mellitus, which may increase the risk of cancer [10, 11]. Second, the patient’s family members were chronic, heavy cigarette smokers [12]. Third, the patient’s younger brother had colon cancer [13]. Synchronous quadruple MPCs are extremely rare, and all such cancers can be found in the early stage. MPCs in patients with oral cavity cancer mainly occur in the HNLE region [6]. In contrast, the incidence of non-HNLE cancer, including cancer of the kidney or breast, is relatively low in patients with oral cavity cancer [6]. The 1973–2000 SEER Cancer Registries indicates that of 26,984 patients with oral cavity cancer, the rate of second primary kidney cancer and breast cancer occurring within 1 year after oral cavity cancer diagnosis was only about 0.03 and 0.11 %, respectively [14]. There are few reports of oral-cavity–kidney combinations of MPCs [1, 2, 15, 16]. We found no other English-language literature describing synchronous MPCs as oral-cavity–kidney combinations [9, 17–19].

The second important clinical issue illustrated by this case report is that contrast-enhanced CT should cover an extended region ranging from the liver to the inferior margin of the kidney in patients with oral cavity cancer to avoid overlooking kidney MPC. We usually perform neck, chest, and liver CT scans to detect MPCs or distant metastases in patients with oral cavity cancer. In these cases, we must evaluate the MPCs or distant metastases with nothing but contrast-enhanced CT. We performed a liver CT scan containing the kidneys and incidentally noticed an early-stage mass of the right kidney. In patients with oral cavity cancer, the probability of detecting kidney cancer is low according to the 1973–2000 SEER Cancer Registries [14]. However, recent reports have indicated that the lifetime risk for developing kidney cancer is about 1.6 % [20] and that the incidence of RCC has continued to increase [21]. Furthermore, early-stage RCC can remain clinically silent for a long time, and the clinical symptomatology is generally expressed in patients with advanced disease [22]. Advanced or metastatic RCC is associated with a much lower 5-year disease-specific survival rate than is early-stage RCC [23]. According to the lack of symptoms and the invisibility with FDG-PET, hidden synchronous renal cancer in patients with oral cavity cancer may be much more frequently present than previously thought. Therefore, clinicians should detect the kidney cancer in the early stage using an extended CT scan with consideration of both the benefits and risks, the latter of which include high cost and radiation exposure. The risk of development of secondary kidney or breast cancer after oral cavity cancer is not particularly higher than the risk of after other HNLE or skin (non-melanoma) cancers [14, 24–27]. Therefore, in patients with not only oral cavity cancers but also other HNLE or skin cancers, hidden kidney cancer may be detected by CT covering an extended region ranging from each routine region to the inferior margin of the kidney.

In general, the performance of several types of examinations can help to prevent overlooking synchronous MPCs. FDG-PET is usually used to identify synchronous MPCs; however, FDG-PET sometimes identifies false-positive lesions. In the current case, we could not detect the kidney mass by FDG-PET. The application of FDG-PET to the urinary tract is relatively limited because this tract is the major excretion route for FDG, and background activity may thus obscure the presence of lesions [28]. Additionally, FDG-PET is generally expensive; therefore, some patients with oral cavity cancer are denied this examination before cancer therapy.

## Conclusions

The combination of MPCs described herein has not been reported to date. To avoid overlooking kidney MPC, we suggest that contrast-enhanced CT extends to the inferior margin of the kidney in patients with oral cavity cancer. Early-stage RCC can remain clinically silent for a long time; therefore, this kidney cancer in patients with oral cavity cancer may be overlooked. Further reports should be accumulated to make a new protocol for the CT scan range in patients with oral cavity cancer to detect hidden synchronous renal cancer, which may be much more frequently present than previously thought.

Importantly, we followed up this patient for 5 years after surgery. The patient was alive and well with no clinical or radiologic signs of recurrent or metastatic disease at the time of writing.

## Consent

Written informed consent was obtained from the patient for publication of this case report. A copy of the written consent is available for review from the Editor-in-Chief of this journal.

## Abbreviations

CT: computed tomography
FDG-PET: 2-[18F]-fluoro-2-deoxy-d-glucose positron emission tomography
HNLE: head and neck, lung, and esophagus
MPCs: multiple primary cancers
RCC: renal cell carcinoma
SCC: squamous cell carcinoma
